# The associations between physical activity, sedentary behaviour, and sleep with mortality and incident cardiovascular disease, cancer, diabetes and mental health in adults: a systematic review and meta-analysis of prospective cohort studies

**DOI:** 10.1186/s44167-023-00026-4

**Published:** 2023-09-04

**Authors:** Mitch J. Duncan, Leah Murphy, Stina Oftedal, Matthew J. Fenwick, Grace E. Vincent, Sasha Fenton

**Affiliations:** 1 School of Medicine & Public Health, College of Health, Medicine, and Wellbeing, The University of Newcastle, University Drive, Callaghan, NSW 2308 Australia; 2 Active Living Research Program, Hunter Medical Research Institute, Kookaburra Circuit, New Lambton Heights, NSW 2305 Australia; 3 Queensland Cerebral Palsy and Rehabilitation Research Centre, Faculty of Medicine, The University of Queensland, South Brisbane, QLD 4101 Australia; 4 Appleton Institute, School of Health, Medical and Applied Sciences, Central Queensland University, Wayville, South Australia Australia

**Keywords:** Physical activity, Sleep, Sleep health, Mortality, Prospective, Sitting, Muscle-strengthening

## Abstract

**Background:**

Physical activity, sedentary behaviour and sleep are interrelated and may have a synergistic impact on health. This systematic review and meta-analysis of prospective cohort studies aimed to evaluate the combined influence of different combinations of these behaviours on mortality risk and incidence of cardiovascular disease (CVD), cancer, diabetes, and mental health.

**Methods:**

Four online databases were used to identify studies from database inception to May 2023. Prospective cohort studies that examined how different combinations of physical activity, sedentary and sleep behaviours were associated with mortality and incident cardiovascular disease, cancer, diabetes and mental health in adults were included. Random effects meta-analyses using the Der Simonian and Laird method were conducted.

**Results:**

Assessment of 4583 records resulted in twelve studies being included. Studies were qualitatively summarised and a sub-group of studies (n = 5) were meta-analysed. The most frequent combination of behaviours was duration of leisure time physical activity and sleep (n = 9), with all-cause mortality (n = 16), CVD mortality (n = 9) and cancer mortality (n = 7) the most frequently examined outcomes. Meta-analysis revealed that relative to High physical activity & Mid sleep, High physical activity and Short sleep was not associated with risk of all-cause mortality (RR = 1.05, 95% CI = 0.97, 1.14), however Low physical activity and Short Sleep (RR = 1.42, 95% CI = 1.24, 1.63), Low physical activity and Mid Sleep (RR = 1.30, 95% CI = 1.12, 1.52), High physical activity and Long Sleep (RR = 1.16, 95% CI = 1.01, 1.32), and Low physical activity and Long Sleep were associated with risk of all-cause mortality (RR = 1.63, 95% CI = 1.21, 2.20).

**Conclusions:**

High levels of physical activity may offset all-cause mortality risks associated with short sleep duration. Low levels of physical activity combined with short sleep duration and any level of physical activity in combination with long sleep duration appear to increase mortality risk. Currently there is limited evidence regarding how dimensions of physical activity, sedentary and sleep behaviours other than duration (e.g., quality, timing, type) are associated with future health status.

**Supplementary Information:**

The online version contains supplementary material available at 10.1186/s44167-023-00026-4.

## Introduction

Physical activity, sedentary behaviour and sleep are recognised risk factors for poor physical and mental health [1, 2] and represent movement patterns across a 24-hr period [3–5]. These behaviours are interrelated, as they co-occur in distinct patterns [6] and are co-dependent from a time-use perspective in each 24-period [3, 4, 7]. The need to consider these behaviours jointly as determinants of health is reinforced by several countries that have adopted 24-hr movement guidelines adults [8, 9] and several prominent organisations (i.e., American Heart Association) highlighting sleep as a risk factor for health in addition to physical inactivity and other risk factors [10–12].

Despite the adoption of 24-hr movement guidelines, the potential health effects of different compositions or patterns of physical activity, sedentary behaviour and sleep, or activity-sleep patterns, remains unclear [3]. This is particularly true for adult populations that have more recently been prescribed 24-hr movement guidelines (e.g., Canadian 2020 24-hr Movement Guidelines for Adults) [3, 13]. Several systematic reviews have been conducted that have included studies using specific statistical models (e.g., isotemporal substitution models, compositional data analysis) to quantify activity-sleep patterns and subsequently examine these associations with health outcomes [3, 13−15]. In systematic reviews that required primary studies to use either isotemporal substitution models or compositional data analysis, the vast majority of studies have used cross-sectional designs (i.e., 36/56 studies [13], 7/8 studies [15], 17/20 studies [3]). Due to the reliance on studies using these specific statistical models, available evidence is based only on the time spent in each behaviour. Focussing on the duration of these activities overlooks that physical activity, sedentary and sleep behaviour can be described using multiple dimensions in addition to duration (i.e., Physical Activity: frequency, intensity, type, duration; Sedentary: duration, frequency of breaks in sedentary; Sleep: duration, timing, variability, satisfaction) [16, 17]. Further, other non-time-based dimensions (e.g., sleep quality) are infrequently accounted for in these studies. Additionally, sleep duration has a U-shaped relationship with health outcomes [2], where relative to adults who sleep 7–8 h per night, mortality risks are increased among adults with shorter or longer sleep durations, which is not accounted for in these prior studies.

Compositional data analyses are one way to quantify different patterns of physical activity, sedentary and sleep behaviour. Other alternative data analysis approaches that may more readily enable multiple or other non-time-based dimensions to be examined are available [4]. One approach is to simply classify each behaviour into groups (e.g., meeting guidelines or not) and then examine associations between joint categories of behaviour and outcomes [4, 18]. A review of studies examining compliance with all three behaviours of the 24-hr guidelines (i.e., number of guidelines adhered to) identified 31 studies, 29 of which were cross-sectional [3]. This is despite the existence of a number of prospective studies examining the relationship between behaviour dyads and health outcomes [19–24]. For example, studies have examined the joint association between the physical activity and short sleep duration [23–26], physical activity and long sleep duration [23–26], physical activity and sleep difficulties [20], physical activity and sleep quality [23], and sedentary behaviour and sleep problems [21]. These studies suggest that physical activity moderates the association between sleep and health outcomes, however, associations are inconsistent across studies [19, 20, 22]. Although these studies only examine behavioural dyads they may provide insight into how physical activity, sedentary and/or sleep behaviours jointly influence health outcomes. This information may be useful to inform the next iteration of 24-hr movement guidelines and future interventions targeting these behaviours. However, to our knowledge no systematic review of this literature has been conducted. Therefore, the aim of this systematic review was to synthesis evidence on the prospective associations of activity-sleep patterns with mortality risk and the incidence of cardiovascular disease (CVD), cancer, diabetes, and mental health in adults.

## Methods

### Protocol registration and reporting

The review protocol was posted on the Open Science Framework (available from https://osf.io/67nw8/) [27]. The review was conducted and reported in accordance with the Preferred Reporting Items for Systematic Reviews and Meta-Analyses (PRISMA) statement [28]. This review used previously published data for all analyses and did not include human participants therefore no informed consent was sought from participants nor was the study approved by a institutional human ethics research committee.

### Eligibility criteria

The Population, Intervention/Exposure, Comparison, Outcomes, Study design (PICOS) framework was used to guide the identification and selection of studies.

### Population

Studies were eligible if they included adults aged ≥ 18 years. For studies that examined the incidence of a disease/condition (e.g., cancer, depression) as the outcome, participants had to be free of the condition at baseline.

### Intervention/exposure

The intervention/exposures of interest were different combinations of either: physical activity and sleep, sedentary behaviour and sleep, or physical activity, sedentary behaviour, and sleep (i.e., High Active/Good Sleep Quality, High Active/Poor Sleep Quality, Low Active/Good Sleep Quality, Low Active/Poor Sleep Quality). As physical activity, sedentary behaviour and sleep can be conceptualised using multiple dimensions (i.e., activity: frequency, intensity, type, duration; Sedentary: duration, frequency of breaks in sedentary; Sleep: duration, timing, variability, satisfaction) [16, 17], studies which measured behaviours using any of these dimensions were included. The exposures could be assessed by either self-report, objective measures (e.g., accelerometry, polysomnography (PSG)), or through clinician assessment. Additional indicators of sleep included symptoms of insomnia (e.g., present/ absent) or obstructive sleep apnoea (OSA) (e.g., present/ absent) using self-report instruments, clinician diagnosis or objective measurement [29]. Studies were not required to use a specific classification of an individual behaviour (e.g., compliance with the physical activity guidelines, or number of categories).

### Comparators

Comparators were different combinations of activity-sleep patterns, and studies needed to compare different classifications of the exposure using a single reference category. Studies were also included that examined the association between changes in activity-sleep patterns over time with health outcomes (i.e., change from High Active/Short Sleep to Low Active/Short Sleep).

### Outcomes

Outcomes of interest were all-cause mortality, cause-specific mortality, and the incidence of any of the following health outcomes: CVD, cancer, diabetes, or mental health. Outcomes were selected based on evidence that the exposures are individually associated with these outcomes, plausible overlapping biological pathways between the individual exposures and these behaviours and knowledge of studies assessing joint associations between exposures and these outcomes [1, 2, 30−34]. Mortality outcomes needed to be assessed by medical record/data linkage, and incident outcomes could be assessed via self-report, clinician assessment, medical record/data linkage or an established screening instrument. Mental health outcomes included symptoms of depression and anxiety. The association between activity-sleep patterns and outcomes had to be reported using adjusted or unadjusted odds ratio, hazard ratio, or relative risk and 95% confidence intervals, or reported data in a way that allowed calculation of odds ratios/relative risk.

### Study Design

Eligible studies were prospective observational cohort studies, with at least a 12-month follow-up [32]. This was required to limit the potential of reverse causality. There were no restrictions imposed on study sample sizes.

#### Exclusion criteria

Studies were excluded if they: (1) included other lifestyle behaviours (e.g., diet) or health characteristics (e.g., use of medication) as part of the joint exposure categories, (2) were cross-sectional, case-control studies, reviews, intervention studies, or protocols. Studies using isotemporal substitution were also excluded, (3) were not reported in the English language, and (4) were not published peer-reviewed articles. If ≥ 2 studies reported the data of the same cohort, the study with the most recent follow-up period was included. No studies were excluded based on risk of bias assessment (see Risk of Bias).

### Search Strategy

Four electronic databases (SCOPUS, CINAHL, MEDLINE and EMBASE) were initially searched from inception to 8 Feburary 2021, and then updated in 1 May, 2023. The predefined search strategy was developed by LM and MJD and agreed to by all authors (Supplementary Tables 1–3). Database searches were conducted by LM and SF. In addition, the author’s research libraries were screened to identify potentially eligible studies, reference lists of reference lists of relevant systematic reviews and included articles (backward citation tracking), and articles citing an included study (forward citation tracking via Scopus) were also manually screened, and authors of included studies were contacted to identify any further studies, these search strategies were not documented in the Open Science Framework protocol and was performed to identify as many relevant articles as possible.

### Study selection

Search results were imported and screened using Covidence to automatically remove duplicates (Veritas Health Innovation, Melbourne). Two independent reviewers (MJD and LM/SF) evaluated the title/abstract and full texts of the studies against the pre-specified inclusion/exclusion criteria. Discrepancies were resolved by discussion between the reviewers. Consensus was reached for all included articles.

### Risk of Bias

Risk of bias was assessed using the Newcastle-Ottawa Scale (NOS) [35]. Two reviewers (SO/MJD, SF) independently assessed each study. The NOS uses three domains to evaluate the risk of bias in prospective studies (selection of participants, comparability, and outcome). Selection of participants includes representative of exposed cohort, selection of the non-exposed cohort, ascertainment of exposure, and demonstration that the outcome of interest was not present at the beginning of the study. Comparability includes the comparability of cohorts based on design/analysis. Outcomes includes adequate assessment of outcome, adequate follow-up time, and adequacy of follow-up. A study is awarded one point for each numbered item within the selection and outcome categories, and a maximum of two points may be given for comparability. The total maximum score is nine points. Based on the total score, studies were allocated into one of three quality categories: low (0–3), moderate (4–6), or high (7–9) quality.

### Data extraction

Data extraction was conducted using a data extraction template developed for this review in Microsoft Excel (Office 365, Version 2209). Data were extracted by one reviewer (SF/SO) and checked by a second (MJD). Extracted information included population characteristics (age, sex), sampling methods, measurement and classification of exposures (recall period, method of assessment, dimension assessed (e.g., activity duration, frequency; sedentary time or sitting; sleep duration, quality), comparison/reference group, outcome/s and outcome ascertainment (e.g., self-report, data linkage), study design, follow-up length, statistical analysis (i.e., statistical analysis, covariates included) and associations.

### Data synthesis

An a priori decision was made to summarise results using meta-analysis only if an adequate number (≥ 5) of comparable effect sizes were available [36]. Several studies examined the association between sleep duration and mortality within strata of physical activity or vice versa and were only included in the narrative review [25, 37], and were not included in the meta-analysis as they were not considered comparable to those that used a single exposure category [18, 23, 24, 26, 38]. Studies used a number of different criteria to classify physical activity and sleep duration including several intermediate levels of either physical activity (e.g., inactive, meeting aerobic guidelines only, meeting muscle strengthening guidelines only, meeting neither guideline; ≤7.5 METs, 7.5–14.9 METs, 15.0-29.9 METS; ≥30.0 METs) or sleep (e.g., <6 h, 6-<8 h, 8-<9 h, ≥ 9 h; <7 h; <6, 6-6.5, 6.6–7.4, 7.5-8.0, > 8.0). Consequently, meta-analysis was undertaken to examine the association between joint categories of physical activity and sleep duration with all-cause mortality using the highest and lowest level of activity and the shortest and longest level of sleep duration.

To account for heterogeneity between studies random effects meta-analyses using the Der Simonian and Laird methodwere undertaken using Stata MP (17) using the meta suite of commands. All studies report Hazard Ratios which were assumed to approximate Relative Risk. To avoid double counting the effect size for mid-sleep/low active in one study that reported the association between short and long sleep duration separately using a common reference group of mid-sleep and high active, the mid-sleep and low active group from the analysis of short sleep duration was omitted [38]. This group was selected as it was a secondary focus and the analysis resulted in larger magnitude effects for mid-sleep and low active [38]. Therefore, it was omitted to provide conservative pooled effects. Meta-analysis was not undertaken for other outcomes due to the limited number of effect sizes available and the heterogeneity between studies. Studies not included in the meta-analysis were narratively summarised as after full text screening was completed, in most cases there was only a single study examining a specific exposure in relation to an outcome.

## Results

### Search results and risk of bias

The search strategy identified 4583 records (Supplementary Fig. 1). After contacting authors, and backwards and forwards citation tracking, an additional five studies were included for screening. After the removal of 1363 duplicates, title and abstract screening was conducted on 3225 articles. From these articles, 3028 were excluded and 197 full texts were screened. Of the 197 studies, 185 were excluded (Supplementary Fig. 1). In total, 12 studies met the eligibility criteria and were included in the review. All included studies were of high quality with a mean score of 7.7 in the Newcastle-Ottawa Scale (Range 7–9) (Supplementary Table 4).

### Description of included studies

Supplementary Table 5 summaries the characteristics of the included studies. The 12 included studies were published between 2014 and 2022 and were conducted in eight countries: USA (n = 5) [18, 24, 37, 39, 40], Australia (n = 1) [41], China (n = 1) [38], Finland (n = 1) [23], Spain (n = 1) [42], Sweden (n = 1) [25], Taiwan (n = 1) [26], and the UK (n = 1) [22]. There was a total of 1,524,584 participants across the included cohorts, with sample sizes ranging from 1638 [23], to 380,055 participants [22] (Supplementary Table 5). Both male and female participants were included in 92% of studies (n = 11), while one study (8%) included only male participants [23]. Of the studies, 58% (n = 7) included adults of all ages (i.e., ≥ 18 years) [18, 23, 26, 37, 38, 40, 41], and 42% (n = 5) included only mid to older aged adults using various criteria (Supplementary Table 5) [22, 24, 25, 39, 42].

### Description of exposures and outcomes

To examine the association between combinations of the separate behaviours, included studies either examined: the association between study outcome/s and sleep duration stratified by physical activity level [25, 37], the association between study outcome/s and sleep duration stratified by sedentary behaviour level [37], a single joint exposure variable comprised of physical activity, sedentary behaviour and sleep [39, 40, 42], physical activity and sleep [23, 24, 26, 38], sedentary behaviour and sleep or presented associations using both stratified and a single joint exposure [18, 22, 41]. The average length of follow-up across the studies ranged from five years [41] to 26 years [23], and one study conducted annual follow-ups of the outcome [41].

**Fig. 1:**
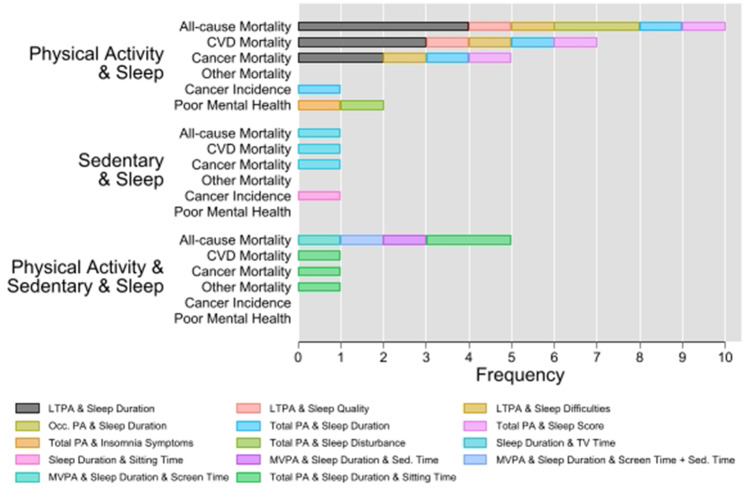
Number of times each combination of exposures has been examined in relation to outcomes

Figure 1 shows the frequency that different combinations of exposures that have been examined across each outcome. One study reported associations stratified by gender [38], and was considered to contribute two associations for each combination of exposure and outcome. Across all combinations of exposures, all-cause mortality was the most frequently examined outcome (n = 16) [18, 22, 24–26, 39, 40, 42], followed by CVD mortality (n = 9) [22, 24–26, 39], and cancer mortality (n = 7) [22, 24–26, 39]. Leisure time physical activity, sleep duration, and multi-domain sitting time were the most frequently examined domains (Fig. 1). The most frequently examined combination of behaviours was leisure time physical activity (LTPA) and sleep duration (n = 9), followed by occupational physical activity and sleep duration (n = 2). All other combinations were examined once only (Fig. 1).

Studies that examined physical activity classified physical activity levels into four [18, 22, 26], three [25, 41] or two levels [23, 24, 37, 38, 40] (Supplementary Table 5). Studies used either five [25], four [26], three [18, 22, 26, 37, 38] or two [23, 24, 40, 41] levels to classify sleep (Supplementary Table 5). Studies that examined sedentary behaviour used four [37], or two [24, 40] levels. Two studies did not classify activity, sleep or sedentary behaviour into any levels [39, 42] instead using statistical techniques (i.e., factor analysis, generalized additive models) [39, 42] to create composite scores which were then classified into multiple levels. The specific criteria used to classify behaviours varied considerably across studies as shown in Supplementary Tables 5, with some using criteria that included at least one level that aligned with minimum compliance to physical activity [18, 22, 23, 40], sleep duration [18, 40] sedentary behaviour guidelines [40]. However, several studies classified behaviours in ways that didn’t appear to align with any specific recommendations for physical activity [24], sleep duration [38, 25]. The single study examining sleep quality did not detail the rationale for the classification used [23]. A single study specifically examined muscle strengthening activity as a separate exposure group [18], although two studies included some measure of muscle strengthening in their assessment of physical activity [39]. Supplementary Table 6 provides the association between each exposure and each outcome for all included studies.

### Qualitative summary

Bayan-Bravo et al., used principal components analysis to identify patterns of physical activity, sedentary behaviour, and sleep duration. Two behavioural patterns were identified, one characterised by low levels of physical activity, high levels of sedentary behaviour and long sleeping time (“sedentary and non-active”); a second, characterised by higher levels of physical activity and low sedentary behaviour without any sleep duration pattern (“active and non-sedentary”). Relative to quartile 1 (i.e., least sedentary) of the sedentary and non-active pattern, only quartiles three and four were associated with increased *all-cause mortality risk*, indicating that lower activity levels, higher sedentary and longer sleep durations are associated with increased *all-cause mortality risk*. Relative to quartile 1 (i.e., most active) of active and non-sedentary pattern, only quartiles three and four were associated with reduced *all-cause mortality risk* (quartile 3: HR = 0.83; quartile 4: HR = 0.68), indicating that higher activity levels and lower higher sedentary behaviours are associated with reduced mortality risk.

Using five sleep duration categories (< 6, 6-6.5, 6.6–7.4, 7.5-8.0, > 8.0) Bellavia et al., examined the association between sleep duration with *all-cause mortality risk* stratified across physical activity tertiles. Across all physical activity tertiles, relative to mid (6.6–7.4) sleep duration the shortest sleep duration category was associated with increased *all-cause mortality risk* (HR = 1.21–1.48), and risks increased with decreasing physical activity levels. Only the longest sleep duration (> 8.0) was associated with increased mortality risk in the lowest tertile of activity (HR = 1.24).

Clarke et al., examined compliance with the Canadian 24-hour movement guidelines for physical activity, sedentary and sleep behaviour by examining the number of behaviours an individual met the guidelines for (i.e., 0–3 guidelines met). Relative to not meeting any guidelines, there were inconsistent associations observed between the number of guidelines met and *all-cause mortality risk* with some evidence that a greater number of guidelines met was associated with reduced mortality risk, and these associations appeared to depend on if accelerometer or self-reported screen time was assessed.

Duncan et al., [43] examined the joint associations between joint categories of physical activity and insomnia symptoms and the onset of *poor mental health* based on symptoms of depression and anxiety. Relative to the High Physical Activity and No Insomnia Symptoms group, any activity level in combination with insomnia symptoms was associated with increased odds of *poor mental health*, as was low physical activity in combination with no insomnia symptoms. Among those with insomnia symptoms, the odds of *poor mental health* increased with decreasing physical activity levels [43]. A similar pattern of observations was also observed when examining joint exposures of physical activity and sleep disturbance which was classified as the presence of insomnia symptoms and short sleep duration (< 7 h per night) (Supplementary Table 5).

Huang et al [22]. examined the joint associations between physical activity and a composite sleep score comprised of sleep duration, chronotype, insomnia symptoms, snoring and daytime sleepiness with the risk of *all-cause mortality*, cancer, CVD coronary heart disease, haemorrhagic stroke, ischaemic stroke, and lung cancer related mortality. Relative to the High Physical Activity and Healthy Sleep Score, the risk of *all-cause mortality* increased with poorer sleep across all physical activity levels and the risks were greatest among adults classified as doing no physical activity. This pattern of associations was relatively consistent when examining CVD mortality and total cancer mortality, however there was no discernible pattern when examining coronary heart disease, haemorrhagic stroke, ischaemic stroke, and lung cancer, where only the combination of no physical activity and poor sleep was consistently associated with increased risk.

Keadle et al., [39] examined the association between a physical behaviour score and the risk of *all-cause, CVD, cancer and other mortality* (Supplementary Table 1). The overall physical behaviour score was classified into quintiles, and relative to the lowest (i.e., poorest behaviour) the risks of all outcomes examined reduced with increasing quintiles of behaviour. This suggests that as overall behaviour improved, mortality risks reduced. Keadle et al., [39] also examined associations between a 10-unit increase in the physical behaviour score and *all-cause mortality* stratified by sex, age category (i.e., younger, older based on median split), self-rated health status (Excellent, Very Good, Good, Fair) and BMI (obese, overweight, normal weight), showing that across all strata a 10-unit increase was associated with reduced mortality risk.

Shen et al., [37] examined the association between categories of sleep duration per night (< 6 h, 6-<8 h, 8-<9 h, ≥ 9 h) and *cancer incidence* stratified across physical activity levels (Low, Medium/High) and also stratified by sitting time (< 2 h, 2–4 h, 4–6 h, > 6 h). Relative to 8-<9 h of sleep, there was no association between any sleep duration category among the Low active group, and only the shortest (< 6 h; HR = 2.32) and longest (≥ 9 h; HR = 2.10) sleep durations were associated with *cancer incidence* among the Medium/High active group. Within the four strata of sitting time, only the shortest (< 6 h; HR = 1.65) sleep duration was associated with *cancer inciden*ce in the < 2 h sitting time.

### Quantitative summary

**Fig. 2:**
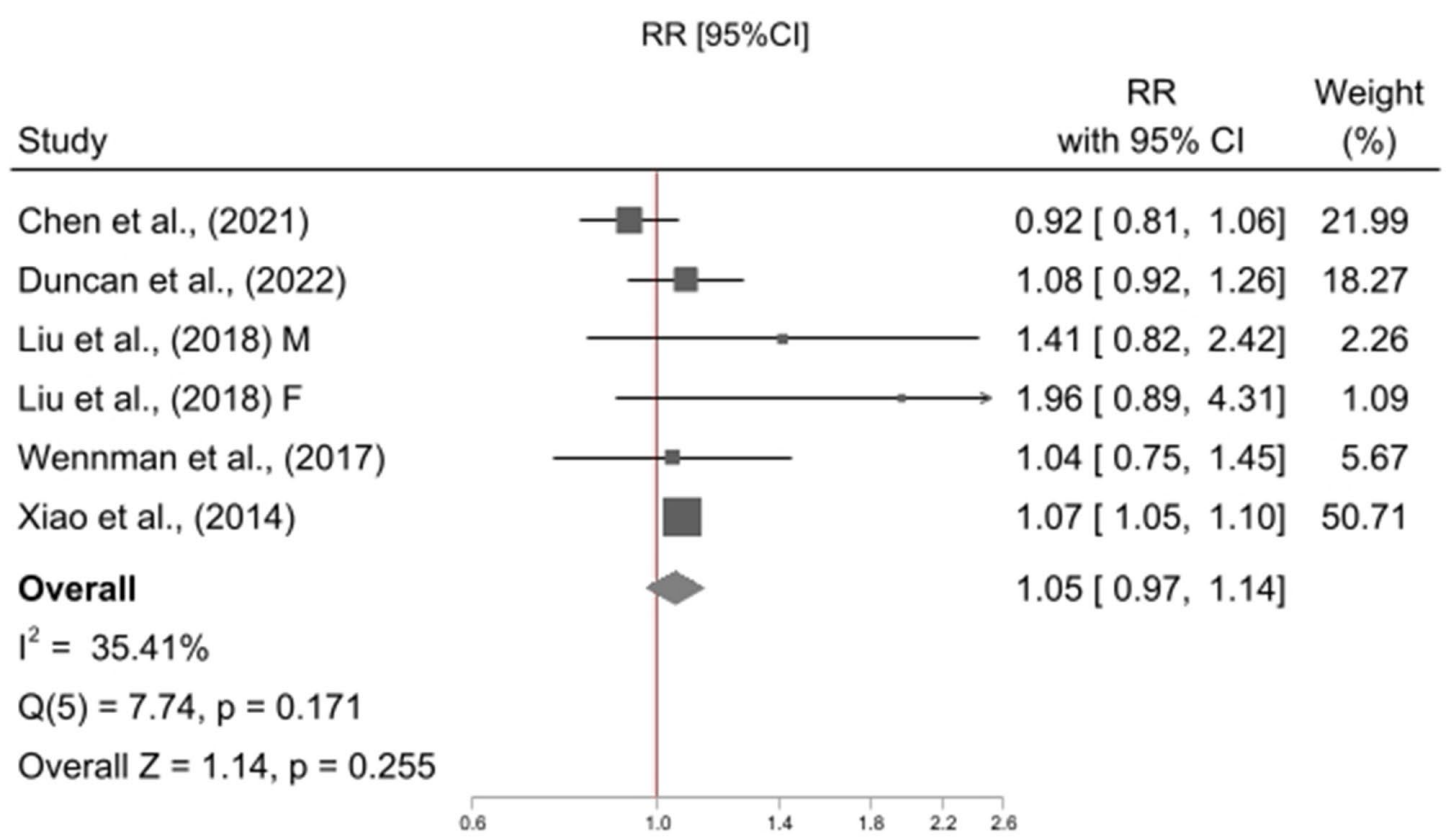
Meta-analysis of high physical activity & short sleep duration relative to high physical activity & mid sleep duration with all-cause mortality

**Fig. 3:**
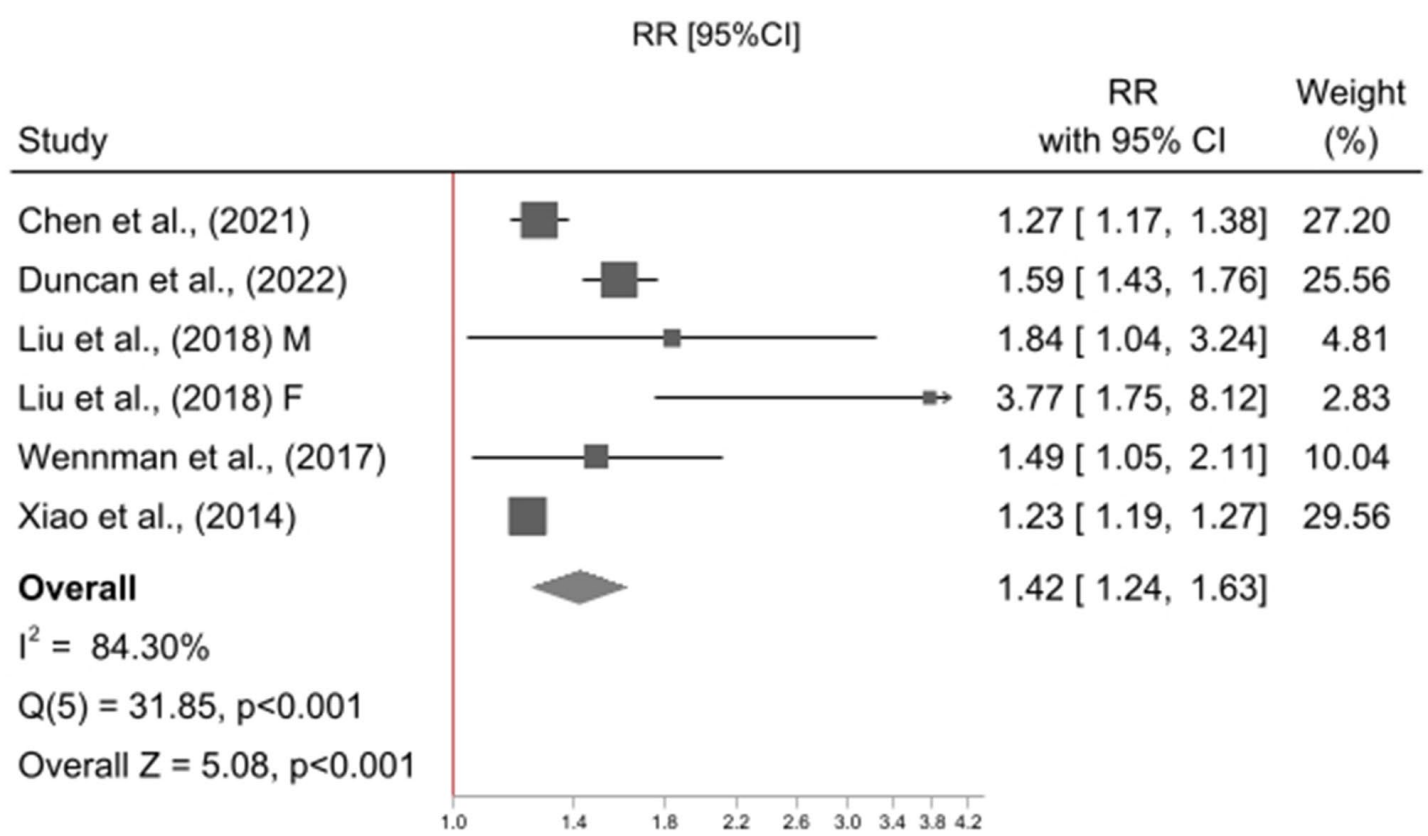
Meta-analysis of low physical activity & short sleep duration relative to high physical activity & mid sleep duration with all-cause mortality

**Fig. 4:**
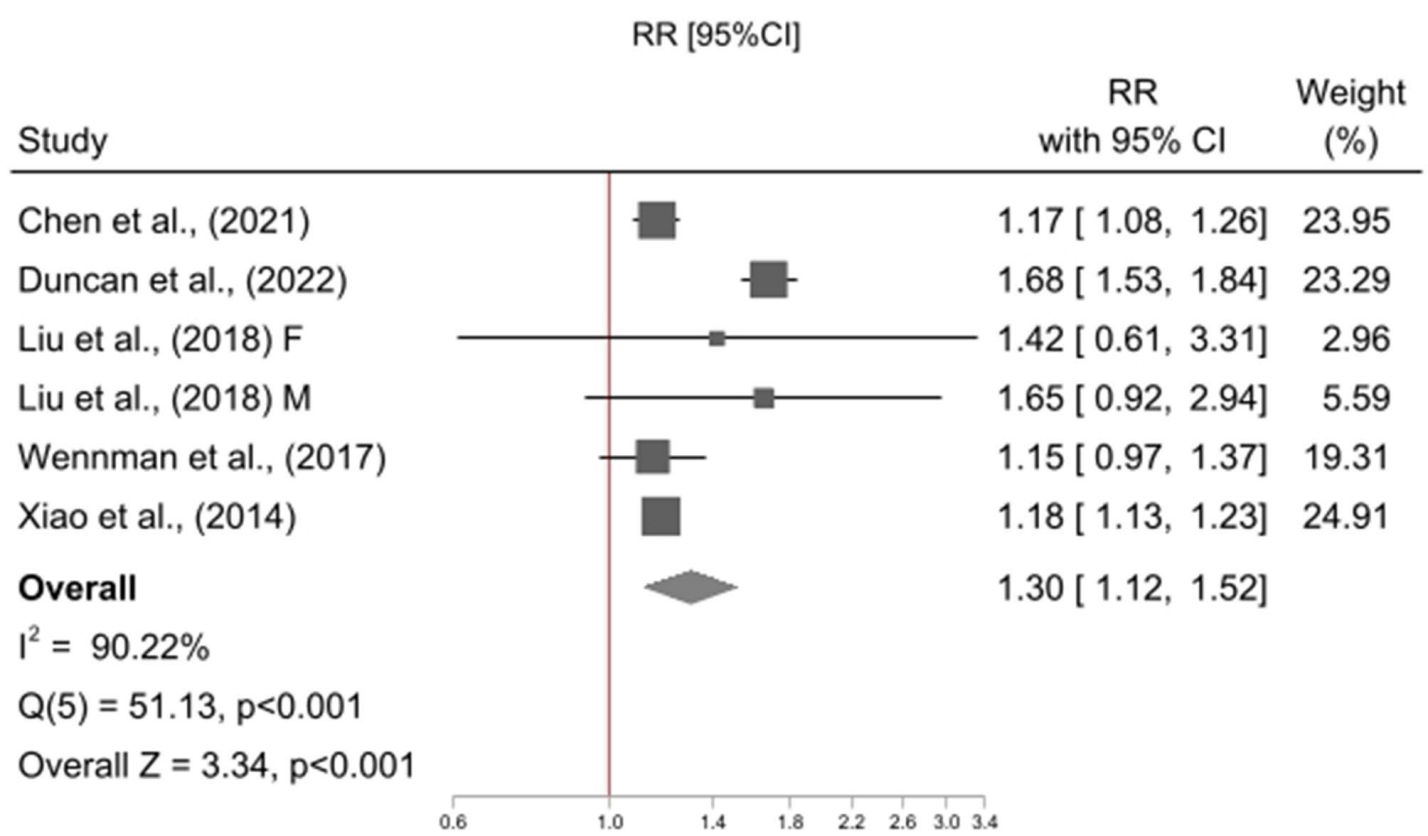
Meta-analysis of low physical activity & mid sleep duration relative to high physical activity & mid sleep duration with all-cause mortality

**Fig. 5:**
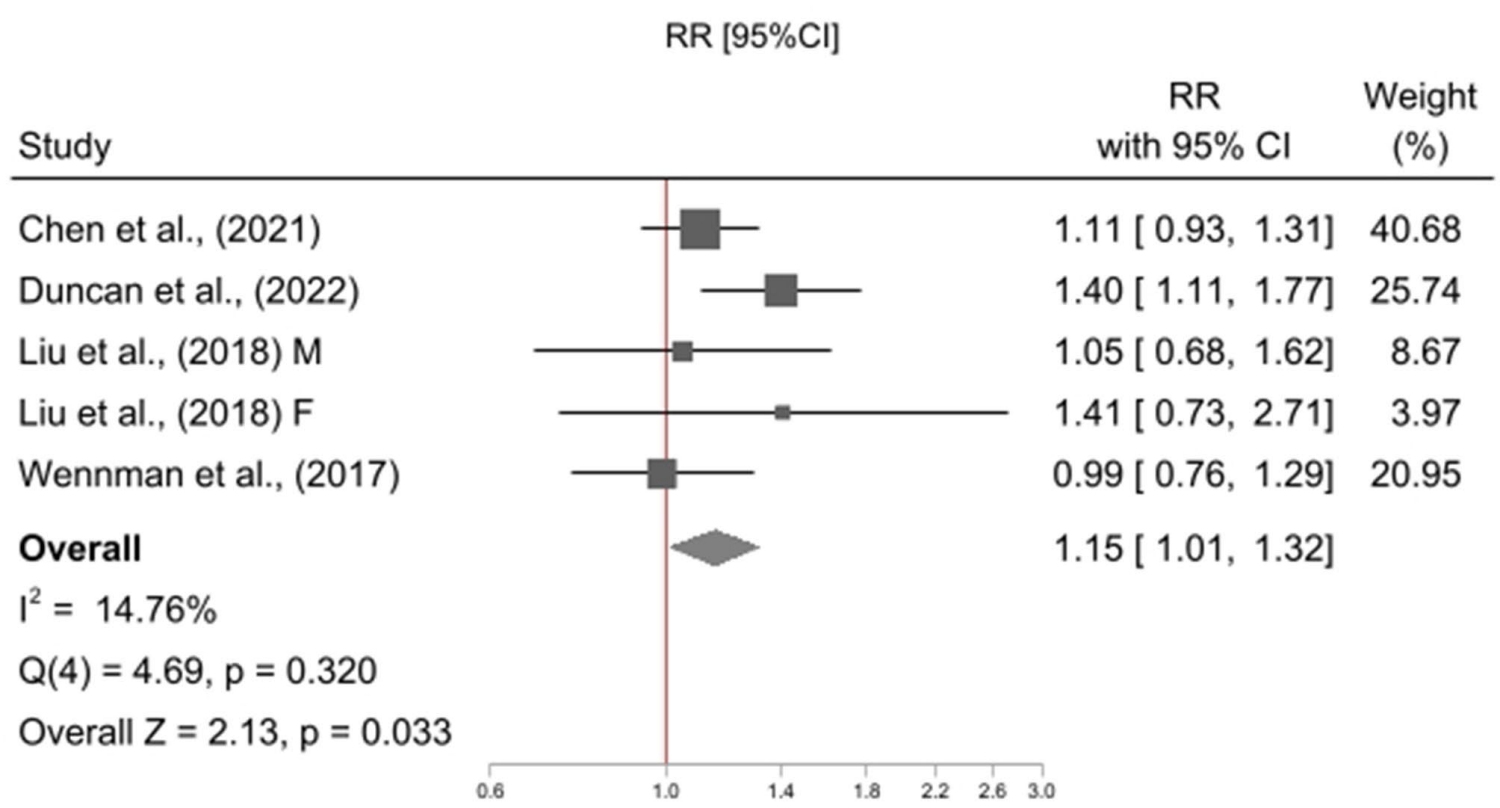
Meta-analysis of high physical activity & long sleep duration relative to high physical activity & mid sleep duration with all-cause mortality

**Fig. 6:**
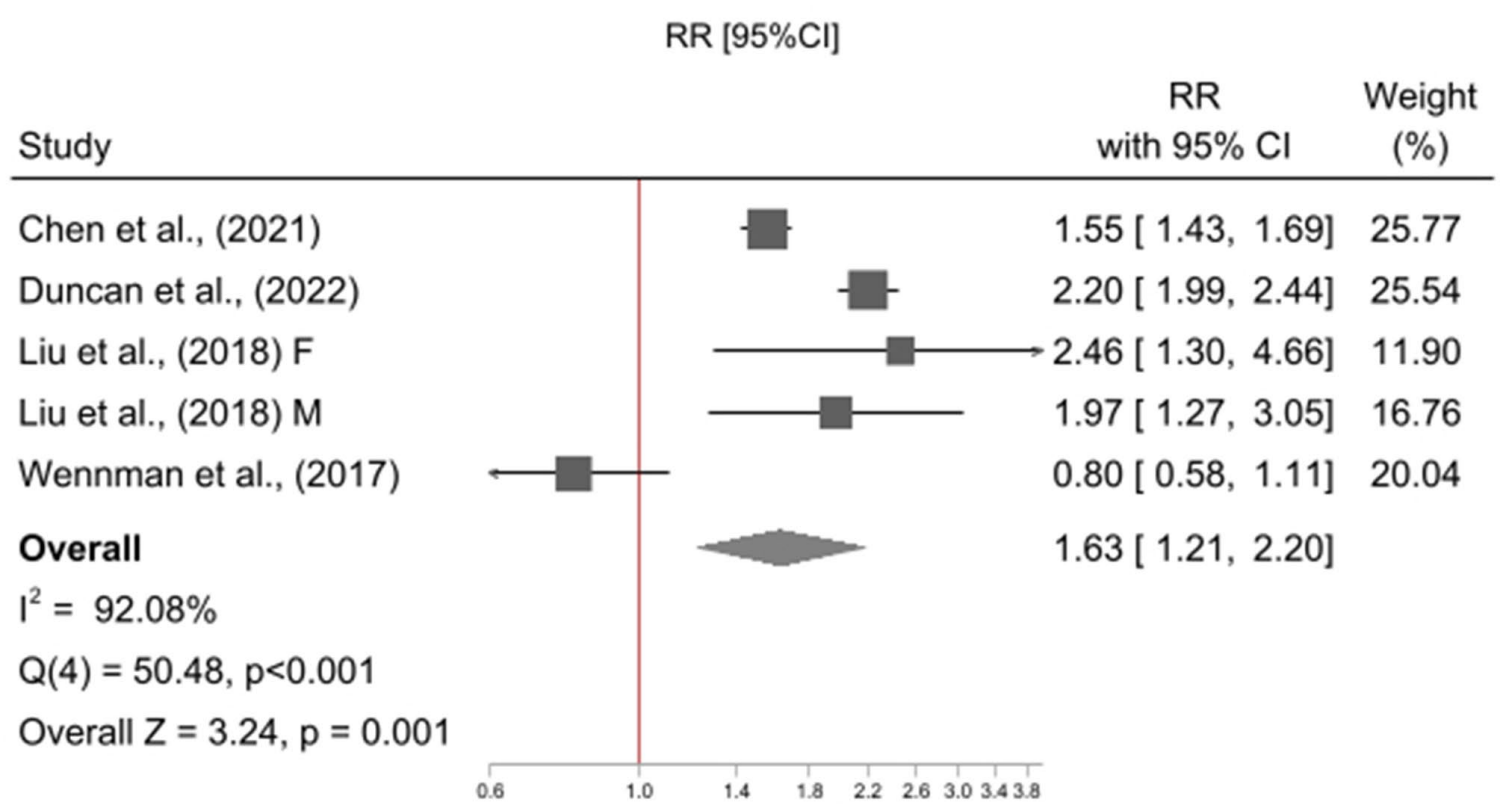
Meta-analysis of low physical activity & long sleep duration relative to high physical activity & mid sleep duration with all-cause mortality

**Fig. 7:**
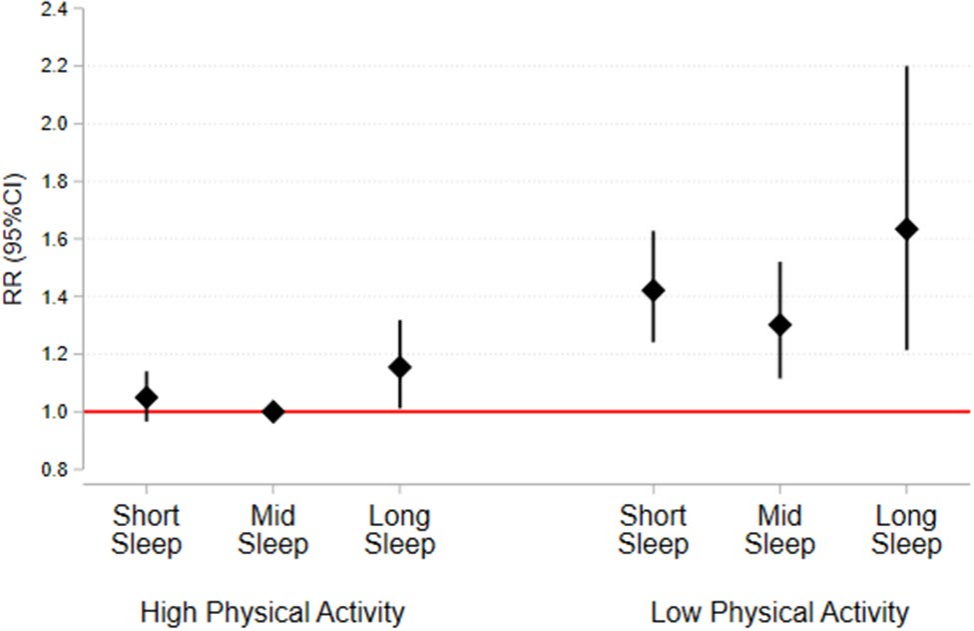
Summary of pooled effect sizes from meta-analyses of physical activity and sleep duration with all-cause mortality

The meta-analysis included five studies [18, 23, 24, 26, 38], contributing a total of six effect sizes as one study reported associations stratified by gender [38]. All studies used a reference category of “high physical activity and mid sleep duration” (High PA & Mid Sleep), and examined physical activity in combination with short sleep duration or mid sleep duration, only four studies [18, 23, 26, 38] examined physical activity in combination with long sleep duration. Relative to High PA & Mid Sleep, High PA and Short Sleep was not associated with risk of all-cause mortality (RR = 1.05, 95% CI = 0.97, 1.14; p = 0.255; I^2^ = 35%; N = 6; Fig. 2) however Low PA and Short Sleep (RR = 1.42, 95% CI = 1.24, 1.63 p = < 0.001; I^2^ = 84%; N = 6; Fig. 3), Low PA and Mid Sleep (RR = 1.30, 95% CI = 1.12, 1.52 p = < 0.001; I^2^ = 90%; N = 6; Fig. 4), High PA and Long Sleep (RR = 1.16, 95% CI = 1.01, 1.32 p = 0.033; I^2^ = 14%; N = 5; Fig. 5), and Low PA and Long Sleep were associated with risk of all-cause mortality (RR = 1.63, 95% CI = 1.21, 2.20 p = 0.001; I^2^ = 92%; N = 5; Fig. 6). Figure 7 illustrates the pooled estimates for each exposure category.

## Discussion

The systematic review summarised prospective cohort studies that examined the association between joint categories of physical activity and sleep, sedentary behaviour and sleep or physical activity, sedentary and sleep with the risk of diabetes, CVD, poor mental health, and mortality in adults. Results of the meta-analysis suggest that high levels of physical activity may offset all-cause mortality risks associated with short sleep durations, yet low levels of physical activity in combination with either short, mid or long sleep duration increase all-cause mortality risk, as does high physical activity in combination with long sleep duration. The qualitative summary of studies not included in the meta-analysis also provided some evidence that low physical activity in combination with number of different indicators of poor sleep increased risk of adverse health outcomes. Twelve studies were included; the majority examined associations between physical activity and sleep duration with all-cause mortality risk. The diversity of exposure combinations and outcomes examined limited the ability to draw conclusions on the association between these behaviours and health outcomes.

This review included 12 prospective studies with adults which is an increase on the two prospective studies [44, 45] included in a 2021 review that required studies to examine either compliance with the Canadian 24-hr Movement Guidelines or apply isotemporal substitution analyses [3]. As such this review offers a unique insight on how combinations of physical activity, sedentary behaviour and/or sleep are associated with the risk of future adverse health outcomes. Specifically, the meta-analysis of five studies suggests that relative to the high physical activity and mid sleep duration groups (except for high levels of physical activity combined with short sleep duration) all other combinations of low or high physical activity with short, mid or long sleep duration increase all-cause mortality risk. Notably, low levels of physical activity in combination with either short (RR = 1.42) or long sleep duration (RR = 1.62) are associated with the greatest all-cause mortality risks, whereas high levels of physical activity either fully or partially offset the risks of short and long sleep. These meta-analytic results are consistent with the results of several individual studies included in the meta-analysis [18, 38] and suggests that interventions that target both physical activity and sleep in combination may be warranted [20, 22, 43, 46, 47]. There was some evidence of a U-shaped relationship between sleep duration and all-cause mortality risk which is consistent with evidence from meta-analyses that examine sleep duration as a separate risk factor [30, 31], with the magnitude of these relationships greater among those in the low physical activity strata.

The results of this review highlight several research gaps. Specifically, that most research to date has examined joint combinations between the duration of leisure time physical activity and sleep duration in relation to all-cause mortality risk, and there is limited understanding of how combinations of other dimensions physical activity, sedentary behaviour and sleep are associated with all-cause mortality risk [22–26, 37, 39, 43]. Physical activity (e.g., frequency, intensity, type [aerobic, muscle strengthening activity], domain [recreational, occupational, transport, household]), sedentary behaviour (e.g., type [screen time, reading, educational, occupational]) and sleep (e.g., duration, timing, quality, satisfaction) can be characterised by multiple dimensions [16, 17, 48]. Research examining these behaviours as separate risk factors demonstrates that the different dimensions may have differing effects on health [49–51] but it is unknown how the combinations of these different behavioural dimensions influence health. Given the interdependence of physical activity, sedentary and sleep behaviours [7, 16] where data are available future studies are encouraged to consider activity-sleep patterns that consider all of these behaviours and to use multiple dimensions of each behaviour as exposures. This likely requires integrating these multiple dimensions using approaches that can accommodate variables measured using different metrics (i.e., time, frequency, quality) such as the use of overall composite scores (e.g., [17, 39]) or other methods (e.g., latent class analyses e.g., [16]). This will provide greater understanding of how overall activity-sleep patterns influence health outcomes. Within the range of health outcomes examined in this review, there was limited evidence examining combinations of physical activity, sleep and/or sedentary behaviours and outcomes other than all-cause mortality [22–26, 37, 39, 43]. In most cases, specific combinations of behaviour were examined in relation to health outcomes in a single study. Finally, well established sociodemographic disparities exist in physical activity, sedentary and sleep behaviours [52] and also chronic disease risks [53]. All studies adjusted for a variety of potential sociodemographic confounders and only two studies [38, 39], examined how health risks of specific behavioural combinations varied according to socio-demographic characteristics, which can be useful in identifying higher risk groups and priority intervention groups. Only a single study included device-based measures of behaviours, [40] with remaining studies utilising self-report measures of all behaviours which have inherent limitations.

Limitations of the current review include only including studies published in English, the health outcomes examined, and the exclusion of studies using isotemporal substitution or compositional data analysis approaches to minimise overlap with prior reviews [14, 15]. Studies examining positive aspects of mental health were omitted which is also a limitation of the review. Studies adjusted for a variety of potential confounders in their analyses, however studies that experimentally manipulate physical activity, sedentary and sleep behaviours are needed to more clearly understand how improving overall activity-sleep patterns can influence health. Experimental studies manipulating various dimensions of physical activity, sleep and sedentary behaviour are necessary to improve understanding of how these behaviours jointly influence health and will be particularly useful to help account for potential confounding that may be present in prospective studies (i.e., residual confounding in the relationship between sleep duration and mortality [54]) and inform the development of future public health guidelines. Withstanding these limitations, from the perspective of minimising all-cause mortality risk, it appears beneficial for adults to engage in higher levels of physical activity and also obtain “mid” sleep durations - which likely correspond with other sleep duration recommendations [8, 55]. As noted, earlier, all-cause mortality risks associated with “short” and “long” sleep appear to be reduced among adults engaging in high levels of physical activity. These observations have important potential implications for public health guidelines related to physical activity and sleep. The underlying mechanisms linking combinations of physical activity, sedentary behaviour and sleep with health outcomes examined in this review are unclear, and experimental studies will be useful in clarifying these. Possible pathways linking these patterns of behaviour to these adverse health outcomes likely overlap and include disruptions to the endocrine system, hormones, inflammation and oxidative stress, blood pressure and cardiometabolic health [56–60].

## Conclusions

There is some evidence that higher levels of physical activity may offset all-cause mortality risks associated with short sleep duration, and that mortality risks remain elevated among combinations of low activity and short sleep duration, and any level of activity combined with long sleep duration. There is a paucity of evidence examining other non-duration dimensions of activity, sedentary and sleep behaviours combine to influence future health status. Available evidence is dominated by research using all-cause mortality as the outcome, and as a consequence there is currently limited evidence of how combinations of these behaviours are prospectively associated with risk of other health outcomes.

## Electronic supplementary material

Below is the link to the electronic supplementary material.

Supplementary Material 1

Supplementary Material 2

Supplementary Material 3

## Data Availability

all data used in this study are provide in supplementary materials.
