# Supplementary material for: The associations between physical activity, sedentary behaviour, and sleep with mortality and incident cardiovascular disease, cancer, diabetes and mental health in adults: a systematic review and meta-analysis of prospective cohort studies
Source: J Act Sedentary Sleep Behav. 2023 Sep 4;2:19. doi: 10.1186/s44167-023-00026-4 (PMC11960281; doi:10.1186/s44167-023-00026-4)
Supplement: Supplementary file 3 — Supplementary Material 3 [file 44167_2023_26_MOESM3_ESM.docx]

The associations between physical activity, sleep and sedentary behaviour, with mortality and health outcomes: A systematic review and meta-analysis of prospective cohort studies *Supplementary material*

Supplementary table 1: Scopus search strategy

| **#** | **Searches** |
| --- | --- |
| 1 | ( ( TITLE-ABS-KEY ( "physical activit*" OR exercise OR "aerobic exercise" OR "resistance training" OR "weight training" OR "physically active" OR walking OR fitness OR "active transport" OR "energy expenditure" OR “muscl* strength*” ) ) |
| 2 | ( TITLE-ABS-KEY ( “physical inactivity” OR sitting OR standing OR "television viewing" OR "computer use" OR "screen time" OR sedentar* ) ) ) |
| 3 | ( ( TITLE-ABS-KEY ( ( "prospective study" ) OR ( "observational study" ) OR ( "cohort studies" ) OR ( "longitudinal studies" ) OR ( "follow-up studies" ) ) |
| 4 | ( TITLE-ABS-KEY ( adult* ) ) |
| 5 | ( TITLE-ABS-KEY ( "sleep health" OR "sleep duration" OR "sleep quality" OR "sleep satisfaction" OR "sleep timing" OR "sleep efficiency" OR "sleep difficulty" OR "sleep disturbance" OR alertness OR "daytime sleepiness" OR insomnia OR "early morning awakening" OR "sleep wake disorders" OR "difficulty maintaining sleep" OR "sleep apn?ea" OR "obstructive sleep apn?ea" OR sleep* OR "sleep disorders" OR "sleep latency" OR arousals OR awakenings ) ) |
| 6 | ( ( TITLE-ABS-KEY ( "all-cause mortality" ) OR TITLE-ABS-KEY ( mortal* ) OR TITLE-ABS-KEY ( morbid* ) OR TITLE-ABS-KEY ( death ) OR TITLE-ABS-KEY ( fatal* ) ) ) |
| 7 | ( TITLE-ABS-KEY ( "cardiovascular disease" OR "coronary heart disease" OR "coronary artery disease" OR "coronary diseases" OR "myocardial ischemia" OR stroke ) ) |
| 8 | ( ( TITLE-ABS-KEY ( cancer OR "cancer survivors" OR survivorship OR "disease-free" OR neoplasm OR carcinoma OR tumo?r OR "prostate cancer" OR "colorectal cancer" OR "breast cancer" ) ) |
| 9 | ( TITLE-ABS-KEY ( depress* OR "depressive disorder" OR dysthymia OR anxiety OR anxious OR "mental illness" OR "mental health" ) ) ) |
| 10 | ( TITLE-ABS-KEY ( "diabetes mellitus" OR "type 2 diabetes" OR diabetes OR "non-insulin-dependent mellitus" ) ) |
| 11 | 3 and 4 |
| 12 | 6 or 7 or 8 or 9 or 10 |
| 13 | 1 and 5 |
| 14 | 2 and 5 |
| 15 | 11 and 12 and 13 |
| 16 | 11 and 12 and 14 |
| 17 | 11 and 12 and 1 and 2 and 5 |

Supplementary table 2: Medline and EMBASE search strategy

| **#** | **Searches** |
| --- | --- |
| 1 | (physical activit* or exercise or aerobic exercise or resistance training or weight training or physically active or walking or fitness or active transport or energy expenditure or muscl* strength*).tw. |
| 2 | (physical inactivity or sitting or standing or “television viewing” or “computer use” or “screen time” or sedentar*).tw. |
| 3 | (prospective study or observational study or cohort studies or longitudinal studies or follow up studies).tw. |
| 4 | adult*.tw. |
| 5 | (sleep health or sleep duration or sleep quality or sleep satisfaction or sleep timing or sleep efficiency of sleep difficulty or sleep disturbance or alertness or daytime sleepiness or insomnia or early morning awakening or sleep wake disorders or difficulty maintaining sleep or sleep apn?ea or obstructive sleep apn?ea or sleep* or sleep disorders or sleep latency or arousals or awakenings).tw. |
| 6 | (all-cause mortality or mortal* or morbid* or death or fatal*).tw. |
| 7 | (cardiovascular disease or coronary heart disease or coronary artery disease or coronary diseases or myocardial ischemia or stroke).tw. |
| 8 | (cancer or cancer survivors or survivorship or disease-free or neoplasm or carcinoma or tumo?r or prostate cancer or colorectal cancer or breast cancer).tw. |
| 9 | (dep* or depressive disorder or dysthymia or anxiety or anxious or mental illness or mental health).tw. |
| 10 | (diabetes mellitus or type 2 diabetes or diabetes or non-insulin-dependent mellitus).tw. |
| 11 | 3 and 4 |
| 12 | 6 or 7 or 8 or 9 or 10 |
| 13 | 1 and 5 |
| 14 | 2 and 5 |
| 15 | 11 and 12 and 13 |
| 16 | 11 and 12 and 14 |
| 17 | 11 and 12 and 1 and 2 and 5 |

Supplementary table 3: CINAHL search strategy

| **#** | **Searches** |
| --- | --- |
| 1 | physical activit* or exercise or aerobic exercise or resistance training or weight training or physically active or walking or fitness or active transport or energy expenditure or muscl* strength* |
| 2 | physical inactivity or sitting or standing or “television viewing” or “computer use” or “screen time” or sedentary* |
| 3 | prospective study or observational study or cohort studies or longitudinal studies or follow up |
| 4 | adult* |
| 5 | sleep health or sleep duration or sleep quality or sleep satisfaction or sleep timing or sleep efficiency of sleep difficulty or sleep disturbance or alertness or daytime sleepiness or insomnia or early morning awakening or sleep wake disorders or difficulty maintaining sleep or sleep apn?ea or obstructive sleep apn?ea or sleep* or sleep disorders or sleep latency or arousals or awakenings |
| 6 | all-cause mortality or mortality or morbidity or death or fatal |
| 7 | cardiovascular disease or coronary heart disease or coronary artery disease or coronary diseases or myocardial ischemia or stroke |
| 8 | cancer or cancer survivors or survivorship or disease-free or neoplasm or carcinoma or tumo?r or prostate cancer or colorectal cancer or breast cancer |
| 9 | dep* or depressive disorder or dysthymia or anxiety or anxious or mental illness or mental health |
| 10 | diabetes mellitus or type 2 diabetes or diabetes or non-insulin-dependent mellitus |
| 11 | 3 and 4 |
| 12 | 6 or 7 or 8 or 9 or 10 |
| 13 | 1 and 2 |
| 14 | 2 and 5 |
| 15 | 11 and 12 and 13 |
| 16 | 11 and 12 and 14 |
| 17 | 11 and 12 and 1 and 2 and 5 |

Supplementary figure 1: Flow diagram of article identification and inclusion in the systematic review and meta-analysis

Records identified through database searching
(n = 4583)

**SCREENING**

**INCLUDED**

**ELIGIBILITY**

**IDENTIFICATION**

Additional records identified through other sources
(n = 5)

Duplicates removed
(n = 1363)

Records screened
(n = 3225)

Records excluded
(n = 3028)

Full-text articles assessed for eligibility (n = 197)

Full-text articles excluded, with reasons
(n = 185)

Incorrect exposure (n = 120)

Incorrect outcome (n = 25)

Incorrect study design (n = 20)

Not published peer-reviewed article (n = 14)

Not English language (n = 5)

Duplicate (n = 1)

Studies included in qualitative synthesis
(n = 12 studies)

Studies included in meta-analyses (n = 5 studies)

Supplementary table 4: Risk of bias assessment using the Newcastle-Ottawa Scale for cohort studies

| Study | Selection | | | | Comparability | | Outcome | | | Total quality score |
| --- | --- | --- | --- | --- | --- | --- | --- | --- | --- | --- |
|  | Representativeness of the exposed cohort | Selection of non- exposed cohort | Ascertainment of exposure (method) | Outcome of interest was not present at start of study or baseline assessment | Adjusted for the most important risk factors | Adjusted for other risk factors | Assessment of outcome | Follow-up length | Loss to follow-up rate |  |
| Bayan-Bravo et al., 2019^1^ | 1 | 1 | 0 | 1 | 1 | 1 | 1 | 1 | 1 | 8 |
| Bellavia et al,, 2014^2^ | 1 | 1 | 0 | 1 | 1 | 1 | 1 | 1 | 0 | 7 |
| Chen et al., 2021^3^ | 1 | 1 | 0 | 1 | 1 | 1 | 1 | 1 | 1 | 8 |
| Clarke et al., 2021^4^ | 1 | 1 | 0 | 1 | 1 | 1 | 1 | 1 | 1 | 8 |
| Duncan et al., 2022a^5^ | 1 | 1 | 0 | 1 | 1 | 1 | 0 | 1 | 1 | 7 |
| Duncan et al., 2022b^6^ | 1 | 1 | 0 | 1 | 1 | 1 | 0 | 1 | 1 | 7 |
| Huang et al., 2021^7^ | 1 | 1 | 0 | 1 | 1 | 1 | 1 | 1 | 1 | 8 |
| Keadle et al., 2019^8^ | 1 | 1 | 0 | 1 | 1 | 1 | 1 | 1 | 1 | 8 |
| Liu et al., 2018^9^ | 1 | 1 | 1 | 1 | 1 | 1 | 1 | 1 | 1 | 9 |
| Shen et al., 2019^10^ | 1 | 1 | 1 | 1 | 1 | 1 | 1 | 0 | 0 | 7 |
| Wennman et al., 2017^11^ | 0 | 1 | 0 | 1 | 1 | 1 | 1 | 1 | 1 | 7 |
| Xiao et al., 2014^12^ | 1 | 1 | 0 | 1 | 1 | 1 | 1 | 1 | 1 | 8 |

References

1. Bayán-Bravo A, Pérez-Tasigchana RF, López-García E, Martínez-Gómez D, Rodríguez-Artalejo F, Guallar-Castillón P. The association of major patterns of physical activity, sedentary behavior and sleeping with mortality in older adults. J Sports Sci. 2019;37(4):424-33.

2. Bellavia A, Åkerstedt T, Bottai M, Wolk A, Orsini N. Sleep duration and survival percentiles across categories of physical activity. Am J Epidemiol. 2014;179(4):484-91.

3. Chen LJ, Hamer M, Lai YJ, Huang BH, Ku PW, Stamatakis E. Can physical activity eliminate the mortality risk associated with poor sleep? A 15-year follow-up of 341,248 MJ Cohort participants. J Sport Health Sci. 2022;11(5):596-604.

4. Clarke AE, Carson V, Chaput JP, Colley RC, Roberts KC, Rollo S, et al. Meeting Canadian 24-Hour Movement Guideline recommendations and risk of all-cause mortality. Appl Physiol Nutr Metab. 2021;46(12):1487-94.

5. Duncan MJ, Oftedal S, Kline CE, Plotnikoff RC, Holliday EG. Associations between aerobic and muscle-strengthening physical activity, sleep duration, and risk of all-cause mortality: A prospective cohort study of 282,473 U.S. adults. Journal of Sport and Health Science. 2022.

6. Duncan MJ, Holliday EG, Burton NW, Glozier N, Oftedal S. Prospective associations between joint categories of physical activity and insomnia symptoms with onset of poor mental health in a population-based cohort. J Sport Health Sci. 2022.

7. Huang BH, Duncan MJ, Cistulli PA, Nassar N, Hamer M, Stamatakis E. Sleep and physical activity in relation to all-cause, cardiovascular disease and cancer mortality risk. British journal of sports medicine. 2022;56(13):718-24.

8. Keadle SK, Kravitz ES, Matthews CE, Tseng M, Carroll RJ. Development and Testing of an Integrated Score for Physical Behaviors. Med Sci Sports Exerc. 2019;51(8):1759-66.

9. Liu F, Zhang H, Liu Y, Sun X, Yin Z, Li H, et al. Sleep Duration Interacts With Lifestyle Risk Factors and Health Status to Alter Risk of All-Cause Mortality: The Rural Chinese Cohort Study. J Clin Sleep Med. 2018;14(5):857-65.

10. Shen J, Chrisman M, Wu X, Chow WH, Zhao H. Sleep duration and risk of cancer in the Mexican American Mano-a-Mano Cohort. Sleep Health. 2019;5(1):78-83.

11. Wennman H, Kronholm E, Heinonen OJ, Kujala UM, Kaprio J, Partonen T, et al. Leisure Time Physical Activity and Sleep Predict Mortality in Men Irrespective of Background in Competitive Sports. Progress in Preventive Medicine. 2017;2(6).

12. Xiao Q, Keadle SK, Hollenbeck AR, Matthews CE. Sleep duration and total and cause-specific mortality in a large US cohort: interrelationships with physical activity, sedentary behavior, and body mass index. Am J Epidemiol. 2014;180(10):997-1006.

Supplementary Table 5: Characteristics of the included studies

| Author, year | | Population (country, n; sex; % female; age range; mean age) | | Exposures (Sleep, Physical activity, Sedentary behaviour) | | Exposure combinations | Exposure measurement | | | Comparison/ Reference group | Length of follow up | Outcome/s | Outcome ascertainment/measurement | Results/  Effect size | Covariates |
| --- | --- | --- | --- | --- | --- | --- | --- | --- | --- | --- | --- | --- | --- | --- | --- |
| Bayan-Bravo et al., 2019^1^ | | Spain;  n= 2851;  M: 1063  F: 1815;  64% Female;  65-97 years;  NR | | Sleep duration  MVPA  Multi-domain sedentary behaviour | | Sleep, physical activity, sedentary behaviour | SR questionnaire  Nurse’s Health Study, Health Professionals’ Follow-up Study  Total time spent per day sleeping or lying down  Total time spent per week walking and participating 15 other activities, (1-4, 5-19, 20-59 min per week or -1.4, 1.5-1.9, 2-3.9, 4-6.9, 7-10 hours per week), Total time spent per week on household activities  Total time spent per week in sedentary behaviour | | | Risk of mortality associated with quartiles of adherence to sedentary and non-active pattern  Reference group: Q1, lowest adherence to sedentary + non-active pattern | 11 years | All-cause mortality | National Death Index linkage | HR: 1.71, 95% CI 1.42-2.07  (Q4, greatest adherence to sedentary + non-active pattern)  HR: 0.68, 95% CI 0.57 - 0.82  (Q4, greatest adherence to non-sedentary + active pattern) | Age, sex, education, marital status, living alone, tobacco consumption, alcohol consumption, body mass index, chronic diseases, agility limitation, dependence for instrumental activities for daily living, dependence for activities of daily living, other physical activity-sedentary behaviour-sleep pattern |
| Bellavia et al., 2014^2^ | | Sweden;  n= 70,973;  M: 37,846  F: 33,127;  47% F;  45-83 years;  NR | | Sleep duration  MVPA | | Sleep, physical activity | SR questionnaire  Sleep duration per day, tertiles <6, 6-6.5, 6.6-7.4, 7.5-8 and >8 hours per day;  Average monthly use of sleeping pills  Total time spent per day on work/occupational activities, housework, walking/bicycling, inactive leisure time, and exercising; tertiles of metabolic equivalent hours per day (<39.3, 39.3-44.2, >44.2) | | | Risk of mortality associated with  sleep duration and tertiles of physical activity (MET hours per day)  Reference group: Sleep duration 6.6-7.4 hours per day | 15 years | All-cause mortality  CVD mortality  Cancer mortality | Swedish National Register of Death Causes linkage | All-cause mortality,  HR: 1.48, 95% CI 1.19 - 1.85  (sleep duration <6 hours per day + <39.3 MET hours per day, shortest sleep duration + lowest physical activity tertile)  HR: 1.24, 95% CI 1.11 - 1.39  (sleep duration >8 hours per day + <39.3 MET hours per day, longest sleep duration + lowest physical activity tertile)  CVD mortality,  HR: 1.45, 95% CI 0.85 - 2.22  (sleep duration <6 hours per day + <39.3 MET hours per day,  shortest sleep duration + lowest tertile of physical activity)  HR: 1.41, 95% CI 1.14 - 1.74  (sleep duration >8 hours per day + <39.3 MET hours per day, longest sleep duration + lowest physical activity tertile)  Cancer mortality,  HR: 1.39, 95% CI 0.89 - 2.17  (sleep duration <6 hours per day + <39.3 MET hours per day, shortest sleep duration + lowest tertile of physical activity)  HR: 0.88, 95% CI 0.85 - 1.41  (sleep duration >8 hours per day + <39.3 MET hours per day, longest sleep duration + lowest physical activity tertile) | Sex, age, body mass index, smoking status and pack-years of smoking, alcohol consumption, education level |
| Chen et al., 2021^3^ | | Taiwan;  n=341,248;  M: 164, 750  F: 176,498;  52% female; NR;  39.7 (SD 13.4) years | | Sleep duration  Sleep disturbances  Leisure time physical activity | | Sleep, physical activity | SR questionnaires;  Sleep duration, h/day,  <6, 6-8 or >8 hours  Sleep disturbances in the last month,  response options, slept well, easily awakened, difficulty falling asleep, and use of medication  Leisure time physical activity: Type, intensity and duration of activity, MET-hours per week (<7.5, 7.5-14.9, 15.0-29.9, ≥30.0) | | | 1. Risk of mortality outcomes associated with sleep duration and physical activity (12 combinations)   Reference group: sleep duration 6-8 h/day + highest PA (>30 MET-h/wk   1. Risk of mortality outcomes associated with sleep disturbance and physical activity (16 combinations)   Reference group: absence of sleep disturbances + highest PA (>30 MET-hours per week | 15.0 (SD 3.4) years | All-cause mortality  CVD mortality  Cancer mortality | Chinese cause-of-death register linkage | All-cause mortality,  HR: 1.27, 95% CI 1.17 - 1.38  (sleep duration <6 hours per day + <7.5 MET hours per day, shortest sleep duration + lowest tertile of physical activity)  HR: 1.34, 95% CI 1.21 - 1.48  (difficulty falling asleep + lowest tertile of physical activity)  CVD mortality,  HR: 1.36, 95% CI 1.12 - 1.66  (sleep duration <6 hours per day + <7.5 MET h/day,  shortest sleep duration + lowest tertile of physical activity)  HR: 1.57, 95% CI 1.23 - 2.00  (difficulty falling asleep + lowest tertile of physical activity)  Cancer mortality,  HR: 1.02, 95% CI 0.94 - 1.15  (sleep duration <6 hours per day + <7.5 MET hours per day,  shortest sleep duration + lowest tertile of physical activity)  HR: 1.15, 95% CI 0.98-1.35  (difficulty falling asleep + lowest tertile of physical activity) | Physical activity, age, sex, education, marital status, smoking, alcohol consumption, fruit and vegetable intake, physical health status |
| Clarke et al., 2021^4^ | | USA;  n= 3471; M: 1756,  F: 1715; 52% female; NR; 46.6 years | | Sleep duration  MVPA  Sedentary behaviour | | Sleep, physical activity, sedentary behaviour | Sleep duration:  SR questionnaire, hours per day, responses rounded to nearest hour  MVPA: accelerometry, 7 days  Sedentary behaviour: accelerometry, 7 days, minutes where activity count was <100,  Screen time: SR questionnaire, hours per day over past 30 days watched TV/videos  Categorised as Yes or No for meeting the Canadian 24-h Movement Guideline recommendations | | | Risk of all-cause mortality associated with meeting or not meeting the Canadian 24-hour Movement Guideline recommendations | 9.4 years | All-cause mortality | National Death Index, Social Security files, and NHANES mortality file linkage | HR: 0.72, 95% CI 0.34 - 1.15  (meeting sleep, physical activity and sedentary behaviour recommendations) | Age, sex, race/ethnicity, education, poverty-to-income ratio, BMI, smoking status, alcohol consumption, diet quality |
| Duncan et al., 2022a^5^ | | USA;  n= 282,473;  M: 82,690  F: 100,101;  55% female;  18-84 years;  NR | | Sleep duration  Leisure time physical activity | | Sleep, physical activity | SR questionnaire  Sleep duration:  Hours per day  Categorised as recommended sleep, short sleep, or long sleep, based on age-specific guidelines from National Sleep Foundation  Leisure time physical activity:  Duration and frequency of light, moderate or vigorous intensity aerobic physical activity per week (≥10 mins in duration);  Number of times performed muscle strengthening activity  Categorised as active, aerobic (AER), muscle strengthening activity (MSA), or inactive | | | Risk of all-cause mortality associated with sleep duration and physical activity (12 combinations)  Reference group:  Active-Recommended (meeting recommended sleep duration + aerobic and muscle strengthening activity guidelines) | 5.4 years | All-cause mortality | National Death Index linkage | HR: 1.08, 95% CI 0.92 - 1.26  (Active-Short)  HR: 1.40, 95% CI 1.11 - 1.77  (Active-Long)  HR: 1.21, 95% CI 1.09 – 1.34  (AER-Rec)  HR: 1.28, 95% CI 1.14 - 1.44  (AER-Short)  HR: 1.54, 95% CI 1.34 – 1.76  (AER-Long)  HR: 1.56, 95% CI 1.36 - 1.80  (MSA-Rec)  HR: 1.43. 95% CI 1.17 – 1.76  (MSA-Short)  HR: 2.32, 95% CI 1.85 – 2.91  (MSA-Long)  HR: 1.68, 95% CI 1.53 – 1.84  (Inactive-Rec)  HR: 1.59, 95% CI 1.43 – 1.76  (Inactive-Short)  HR: 2.20, 95% CI 1.99 – 2.44  (Inactive-Long) | Age, sex, BMI, race and ethnicity, education, employment classification, smoking status, alcohol consumption, self-rated health, prior diagnosis of chronic diseases |
| Duncan et al., 2022b^6^ | | Australia;  n= 10,977;  M: 5298  F: 5679;  52% female;  ≥18 years;  48.8 (16.4) years | | Insomnia symptoms  MVPA | | Sleep, physical activity | SR questionnaire  Insomnia symptoms:   1. Frequency in last month of trouble sleeping 2. Subjective sleep quality   Categorised as insomnia symptoms (trouble sleeping + poor subjective sleep quality), or no insomnia symptoms (no symptoms)  MVPA:  IPAQ-SF, frequency and duration of walking moderate and vigorous intensity physical activity in last week,  MET-minutes and total physical activity per week classified and low physical activity, moderate physical activity or high physical activity categorised | | | Association between insomnia symptoms and physical activity with onset of poor mental health  Reference group:  No insomnia symptoms + high physical activity | 5 years | Poor mental health | Mental Health Inventory (MHI-5) | OR: 1.00, 95% CI 0.89 – 1.13  (No insomnia + moderate physical activity  OR: 1.14, 95% CI 1.01 – 1.29  (No insomnia + low physical activity)  OR: 1.87, 95% CI 1.57 – 2.23  (Insomnia + high physical activity)  OR: 1.93, 95% CI 1.61 – 2.31  (Insomnia + moderate physical activity)  OR: 2.33, 95% CI 1.96 – 2.78  (Insomnia + low physical activity) | Sex, age, marital status, income, education, employment, smoking, alcohol, dietary quality score, BMI, sleep duration, chronic disease |
| Huang et al., 2021^7^ | | UK;  n=380,055;  M: 171,315  F: 208,740;  55% female; NR;  55.9 (SD 8.1) years | | Sleep health  MVPA | | Sleep, physical activity | SR questionnaire  Sleep health: composite score out of 5 based on chronotype, sleep duration, insomnia, snoring and daytime sleepiness. Categorised as healthy sleep (≥4), intermediate (2-3), or poor sleep health (≤1)  Physical activity: International Physical Activity Questionnaire short form.  Categorised as none (0 MET-minutes per week), low (0 to <600 MET-mins per week), medium (600 to <1200 MET-mins per week), and high (≥1200 MET-mins per week) | | | Risk of mortality outcomes associated with sleep health and physical activity (12 combinations)  Reference group: healthy sleep and high physical activity | 11.1 years | All-cause mortality  Total CVD mortality  CVD subtype mortality (coronary heart disease, haemorrhagic stroke, ischaemic stroke)  Total cancer mortality  Lung cancer mortality | Linkage with national datasets from the National Health Service (NHS) Information Centre and NHS Central Register Scotland | All-cause mortality,  HR: 1.57, 95% CI 1.35, 1.82  (poor sleep health + no physical activity)  Total CVD,  HR: 1.67, 95% CI 1.27, 2.19  (poor sleep health + no physical activity)  Coronary heart disease,  HR: 1.59, 95% CI 1.07, 2.37  (poor sleep health + no physical activity)  Hemorrrhagic stroke,  HR: 1.63, 95% CI 0.60, 4.44  (poor sleep health + no physical activity)  Ischaemic stroke  HR: 2.96, 95% CI 1.43, 6.11  (poor sleep health + no physical activity)  Total cancer mortality,  HR: 1.45, 95% CI 1.18, 1.77  (poor sleep health + no physical activity)  Lung cancer mortality,  HR: 1.91, 95% CI 1.30, 2.81  (poor sleep health + no physical activity) | Age, sex, body mass index, socioeconomic status, vegetable and fruit intake, sedentary behaviour, mental health issues, cigarette smoking, employment status, alcohol consumption |
| Keadle et al., 2019^8^ | | USA;  N=163,016  NR;  Q1 55%, Q2 57%, Q3 60%, Q4 61%, Q5 61% female;  NR;  Q1 71 years,  Q2 71 years,  Q3 70 years,  Q4 70 years, Q5 70 years | | Sleep duration  Leisure time physical activity  Multi-domain sedentary behaviour | | Sleep, physical activity, sedentary behaviour | SR questionnaire  Sleep duration, hours per day, categorised as <5 h/day, 5-7 h/day, 7-8 h/day, >9 h/day  Leisure time physical activity: Time spent per week in last 12 months in 16 activities, 0, 15 min, 30 min, 1 h, 1.5 h, 2-3 h, 4-6 h, 7-10 h, >10 h. MET-h/day calculated  Sedentary behaviour:  Hours per day spent sitting in last 12 months, 0, <3 hours, 3-4 hours, 5-6 hours, 7-8 hours, 9-10 hours, 11-12 hours, >12 hours.  Physical behaviour score developed, ranging from 0 to 100, that integrated different types and intensities of sleep, physical activity and sedentary behaviours (0 = highest risk of survival, 100 = lowest risk of survival)  Quintile cut-offs: Q1 0-66.47, Q2 >66.47-73.63, Q3 >73.63-79.03, Q4 >79.03-84.85, Q5 >85.85 | | | Risk of mortality outcomes associated with physical behaviour score (quintiles)  Reference group: Quintile 1 (highest risk of survival) | 6.6 years | All-cause mortality  CVD mortality  Cancer mortality  Other mortality | Social Security Administration Death Master File and the National Death Index linkage | All-cause mortality,  HR: 0.53. 95% CI 0.49, 0.57  (Q5, lowest risk of survival)  CVD mortality,  HR: 0.42, 95% CI 0.37, 0.48  (Q5, lowest risk of survival)  Cancer mortality,  HR: 0.75, 95% CI 0.68, 0.85  (Q5, lowest risk of survival)  Other mortality,  HR: 0.42, 95% CI 0.36, 0.48  (Q5, lowest risk of survival) | Age, sex, education, smoking history, race, overall health, BMI, depression, heart disease |
| Liu et al., 2018^9^ | | China;  n=17,184;  M: 6,788  F: 10,396;  60% female;  ≥18 years;  NR | | Sleep duration  Occupational  physical activity | | Sleep, physical activity | SR questionnaire  Sleep duration: hours per day during past month, <6.5, 6.5-7.5, 7.5-8.5, 8.5-9.5, ≥9.5  Sleep/OSA  Polysomnography  OSA: non-OSA, mild, moderate, or severe  Occupational physical activity: International Physical Activity Questionnaire, categorised as low, moderate, or high | | | Association between sleep duration and physical activity, and all-cause mortality  Reference group: 6.5-7.5 hours of sleep per day | 6 years | All-cause mortality | Death data from interviews with family members and ascertained via the local Centres for Disease Control and Prevention | HR: 1.35, 95% CI 0.88, 2.08  (sleep duration <6.5 hours per day + low physical activity)  HR: 1.46, 95% CI 0.86, 2.47  (sleep duration <6.5 hours per day + high physical activity)  HR: 1.48, 95% CI 1.03 - 2.12  (sleep duration ≥9.5 hours per day + low physical activity)  HR: 1.23, 95% CI 0.81 - 1.87  (sleep duration ≥9.5 hours per day + high physical activity) | Sex, age, marital status, education level, monthly income, smoking status, alcohol, tea drinking, body mass index, systolic blood pressure, fasting plasma, glucose, triglycerides, high-density lipoprotein cholesterol levels |
| Shen et al., 2019^10^ | | USA;  n=10,802;  M: 2165  F: 8637;  80% female;  20-60 years; NR | | Sleep duration  Physical activity (undefined)  Sedentary behaviour | | Sleep, physical activity  Sleep, sedentary behaviour | SR questionnaire (via structured interview)  Physical activity, undefined: categorises as low, medium and high  Sleep duration:  Hours per night, categorised as <6, 6-8, 8-9, ≥9  Sitting time:  Hours per day, <2, 2-4,4-6, >6 | | | Association between sleep duration and cancer risk stratified by physical activity level  Association between sleep duration and cancer risk stratified by sitting time | NR  Followed-up annually | Incidence of cancer | Annual phone call to update on new cancer diagnoses  Confirmed diagnosis via Texas Cancer Registry | HR: 1.16, 95% CI 0.81, 1.65  (sleep duration <6 hours per day + low physical activity)  HR: 0.93, 95% CI 0.62 - 1.41  (sleep duration ≥9 hours per day + low physical activity)  HR: 1.68, 95% CI 0.75, 3.78  (sleep duration <6 hours per day + sitting time >6 hours)  HR: 1.03, 95% CI 0.44 - 2.39  (sleep duration ≥9 hours per day + sitting time >6 hours) | Birthplace, language acculturation, age, sex, marital status, education level, smoking status, drinking status, sitting time, and BMI category |
| Wennman et al., 2017^11^ | | Finland;  N= 1638;  M: 1638;  0% female;  NR;  55 years | | Sleep duration  Sleep quality  Leisure time physical activity | | Sleep, physical activity | SR questionnaire  Sleep duration:  h/day, (≤ 6 hours, 6.5, 7, 7.5, 8, 8.5, 9, 9.5, and ≥ 10,  categorised as short sleep (<6.5 h hours per day), mid sleep (6.5, 7, 7.5, 8, 8.5 hours per day), and long sleep (9, 9.5, ≥10 hours per day)  Sleep quality:  Usual sleep quality, well, fairly well, fairly poor, poorly, cannot say, categorised as good sleep (fairly well and fairly poorly), and Poor sleep  Leisure time physical activity: MET mins per week, categorised as insufficient (<450), and sufficient (>450) | | | Association between sleep duration and all-cause mortality stratified by leisure time physical activity  Association between sleep quality and all-cause mortality stratified by leisure time physical activity | 26 years | All-cause mortality | Population Register of Finland; Statistics Finland | HR: 1.49, 95% CI 1.05, 2.11 (short sleep duration + insufficient physical activity)  HR: 0.80, 95% CI 0.58 - 1.11  (long sleep duration + insufficient physical activity)  HR: 1.46, 95% CI 0.94, 2.27 (poor sleep quality + insufficient physical activity) | FA model: history of sports, SES variables, other lifestyles, sleep medication, chronic disease |
| Xiao et al., 2014^12^ | | USA;  n=239,896;  NR;  <5 hours sleep duration 52% female,  5-6 hours sleep duration 45% female,  7-8 hours sleep duration 43% female,  ≥9 hours sleep duration 45% female;  51-72 years;  NR | | Sleep duration  MVPA  TV watching | | Sleep, physical activity, sedentary behaviour | SR questionnaire  Sleep duration:  h/night in past year, <5, 5-6, 7-8, ≥9,  Dichotomised as unhealthy (≤6 h/night) and healthy (≥7 hours per night)  Duration ≥9 hours per day excluded  Napping during the day: Hours per day in past year, none, <1, 1-2, 3-4, ≥5  Physical activity: frequency/duration of MVPA per week in the last 10 years, never, rarely, <1, 1-3, 4-7, >7, Dichotomised as healthy (≥1 hour per week), and unhealthy (<1 hour per week)  Sitting overall:  h/week in the past year, <3, 3-4;  Television viewing:  Hours per week in the past year, none, <1, 1-2, 3-4, 5-6, 7-8, ≥9, Dichotomised as healthy (≤2 hours per day), and unhealthy (≥3 hours per day) | | | Association between sleep duration, MVPA, TV viewing, and mortality outcomes | 14 years | All-cause mortality  CVD mortality  Cancer mortality | Social Security Administration Death Master File linkage  Cause of death from National Death Index Plus | All-cause mortality:  RR: 1.25, 95% CI 1.19, 1.32 (sleep duration <7 hours per day, MVPA ≤1 hour per week, TV viewing ≥3 hours per day)  CVD mortality:  RR: 1.54, 95% CI 1.38, 1.72 (sleep duration <7 hours per day, MVPA ≤1 hour per week, TV viewing ≥3 hours per day)  Cancer mortality:  RR: 1.18, 95% CI 1.09, 1.27 (sleep duration <7 hours per day, MVPA ≤1 hour per week, TV viewing ≥3 hours per day) | Age, sex, race/ethnicity, marital status, education, self-reported health, smoking status, smoking dose, years since quitting smoking, and alcohol drinking and BMI |
|  |  | |  | |  | | |  |  |  |  |  |  |  |  |

Abbreviations: AHI, Apnea Hypopnea Index; BMI, body mass index; CI, confidence interval; CVD, cardiovascular disease; F, female; HR, hazard ratio; M, male; MET-h, metabolic equivalent hours; min, minutes; MVPA, moderate to vigorous intensity physical activity; NR, not reported; OR, odds ratio; OSA, obstructive sleep apnoea; Q, quintile; REM, rapid eye movement; RR, relative risk; SD, standard deviation; SR, self-reported; TV, television
